# Correlation data of (*Z*)-1-[4-(trifluoromethyl)benzylidene]thiosemicarbazide *via* spectroscopic methods and Density Functional Theory studies

**DOI:** 10.1016/j.dib.2019.104673

**Published:** 2019-10-17

**Authors:** Uwaisulqarni M. Osman, Azieda Syafika N. Farizal, Maisara Abdul Kadir, Mohd Hasmizam Razali, Mohd Zul Helmi Rozaini, Suhana Arshad

**Affiliations:** a Advanced Nano Materials (AnoMa) Research Group, Faculty of Science and Marine Environment, Universiti Malaysia Terengganu, 21030, Kuala Nerus, Terengganu, Malaysia; b Institute of Marine Biotechnology, Universiti Malaysia Terengganu, 21030, Kuala Nerus, Terengganu, Malaysia; c X-ray Crystallography Unit, School of Physics, Universiti Sains Malaysia, 11800, USM Pulau, Pinang, Malaysia

**Keywords:** Synthesis, Thiosemicarbazide, DFT, Gaussian

## Abstract

New compound, namely (*Z*)-1-[4-(trifluoromethyl)benzylidene]thiosemicarbazide was successfully synthesized using thiosemicarbazide with 4-(trifluoromethyl)-benzaldehyde in ethanol solution. The data presented in this articles is related to our research articles entitled “Crystal Structure of (*Z*)-1-[4-(Trifluoromethyl)benzylidene]thiosemicarbazide” (Osman et al., 2017) [1]. This work shows the continue data from experimental spectroscopic measurement which are Fourier Transform Infrared (FTIR) and ^13^C Nuclear Magnetic Resonance (^13^C NMR). Assessment on the correlation with theoretical computational data was also carried out through GaussView 5.0.9 and Gaussian09 software. Molecular Electrostatic Potential (MEP) and Highest Occupied Molecular Orbital–Lowest Unoccupied Molecular Orbital (HOMO-LUMO) were also illustrated.

**Table:** Specifications Table

Subject	Chemistry
Specific subject area	Synthetic chemistry, spectroscopy
Type of data	Table Image Figure
How data were acquired	FTIR equipment Bruker Invenio S; NMR equipment Bruker Avance II 400 MHz; All theoretical computational data were obtained through GaussView 5.0.9 and Gaussian 09 software.
Data format	Raw Analysed
Parameters for data collection	The FTIR and^13^C NMR spectrum were recorded at room temperature. Theoretical computational data was carried out through 6-311G (d,p) basic set with B3LYP DFT method.
Description of data collection	The FTIR spectrum was recorded in range of 4000–400 cm^−1^ using ATR sampling technique. Sample was dissolved in deuterated dimethyl sulfoxide (*d*_*6*_-DMSO) to obtain^13^C NMR spectra. The GaussView 5.0.9 and Gaussian 09 software were carried out using typical personal computer.
Data source location	Universiti Malaysia Terengganu, 21030 Kuala Nerus, Terengganu, Malaysia
Data accessibility	Data is available with this article and in the Cambridge Crystallographic Data Centre (CCDC: 1507979)
Related research article	U. M. Osman, A. N. Farizal, S. Arshad, M. A. Kadir. Crystal Structure of (*Z*)-1-[4-Trifluoromethyl)benzylidene] thiosemicarbazide. X-Ray Structure Analysis Online journal, 33 (2017) 3–4. https://doi.org/10.2116/xraystruct.33.3.

**Table:** 

**Value of the Data** • The mentioned data is useful to synthetic researchers who developing chemical database that specifically related with synthesizing thiomisecarbazide derivatives. • The details in the experimental and theoretical computational data is important to produce thiosemicarbazide derivatives for potential used in polymer electrolytes. • The data obtained from IR and^13^C NMR spectroscopic methods are important in structure elucidation of presence molecule.

## 1 Data

**Fig. 1:**
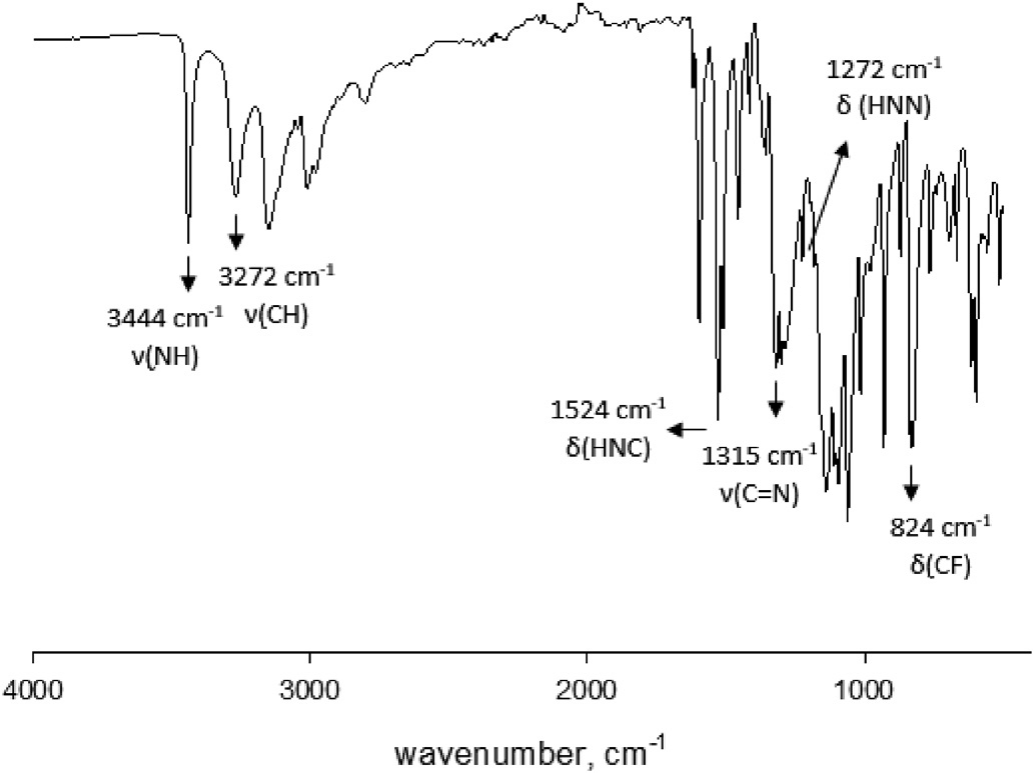
Experimental FTIR spectrum of (*Z*)-1-[4-(Trifluoromethyl)benzylidene]thiosemicarbazide.

**Fig. 2:**
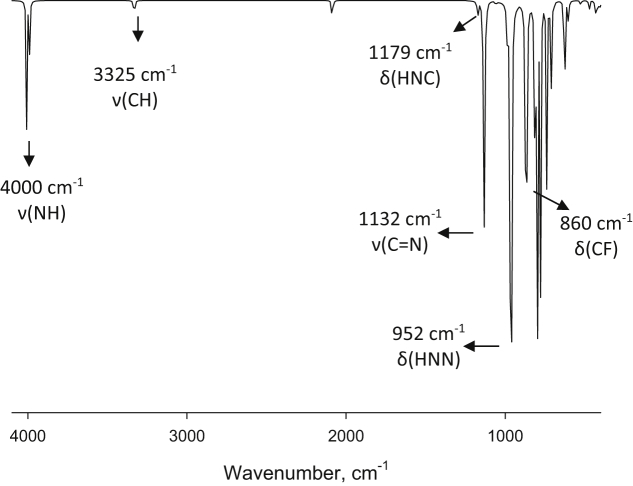
Theoretical FTIR spectrum of (*Z*)-1-[4-(Trifluoromethyl)benzylidene]thiosemicarbazide (Zero-point vibrational energy = 453.584 kJ/Mol).

**Fig. 3:**
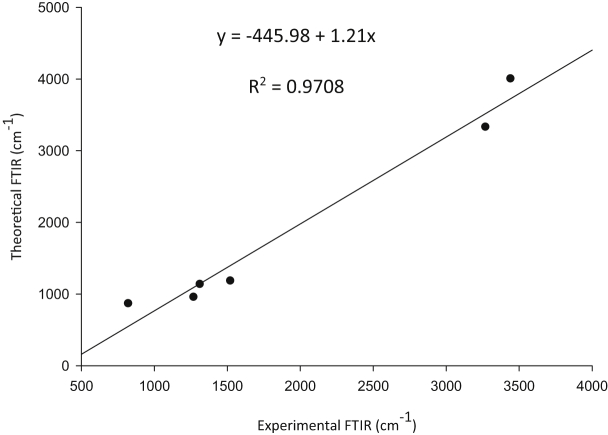
Correlation graphic between experimental and theoretical FTIR of (*Z*)-1-[4-(Trifluoromethyl) benzylidene]thiosemicarbazide.

**Fig. 4:**
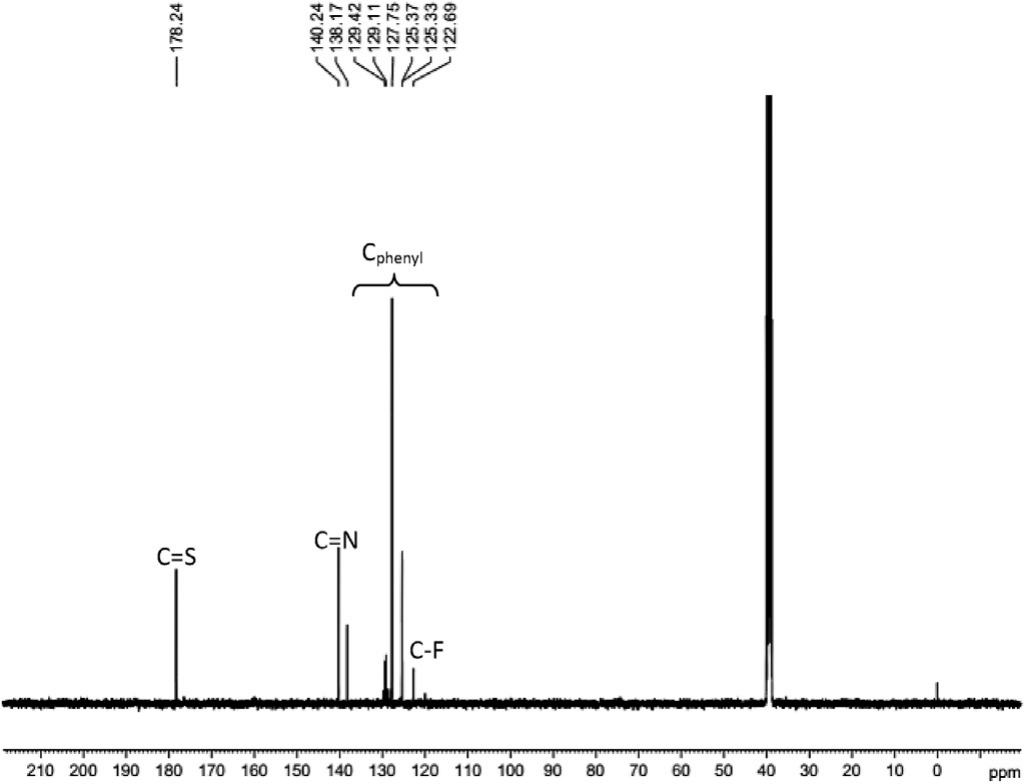
Experimental ^13^C NMR spectrum of (*Z*)-1-[4-(Trifluoromethyl)benzylidene]thiosemicarbazide.

**Fig. 5:**
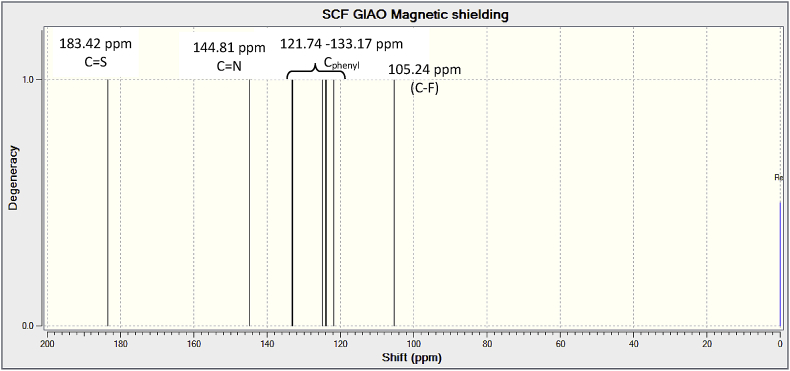
Theoretical ^13^C NMR spectrum of (*Z*)-1-[4-(Trifluoromethyl)benzylidene]thiosemicarbazide.

**Fig. 6:**
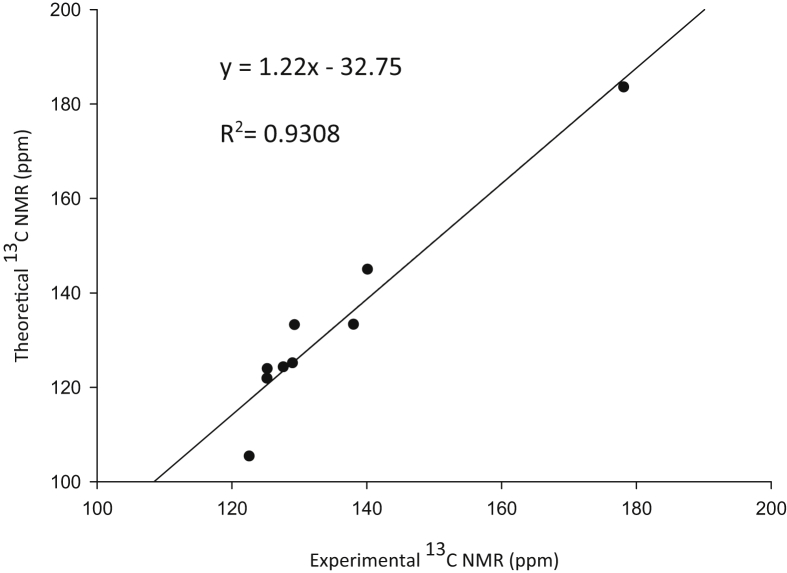
Correlation graphic of experimental and calculated ^13^C NMR of (*Z*)-1-[4-(Trifluoromethyl) benzylidene]thiosemicarbazide.

**Fig. 7:**
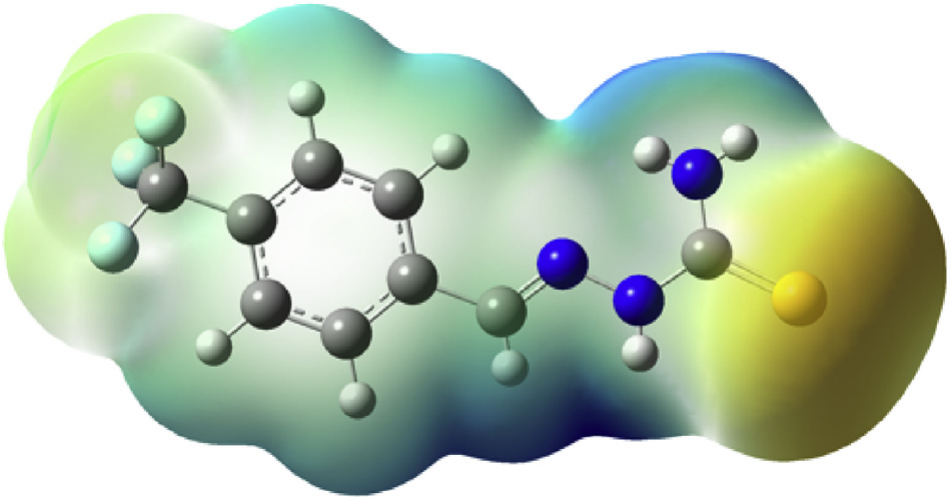
Molecular Electrostatic Potential (MEP) surface diagram of (*Z*)-1-[4-(Trifluoromethyl)benzylidene] thiosemicarbazide.

**Fig. 8:**
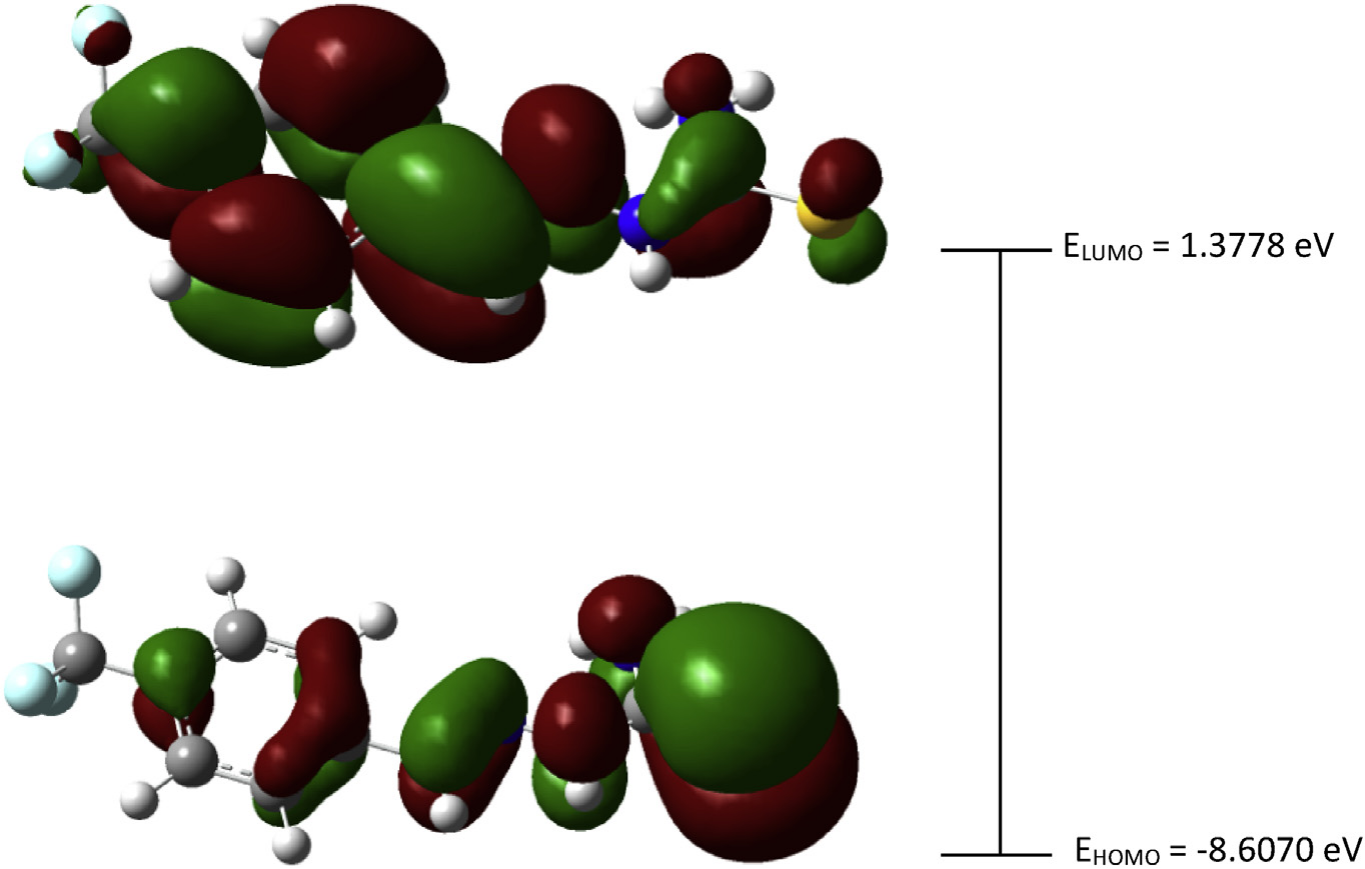
The Highest Occupied Molecular Orbital – Lowest Unoccupied Molecular Orbital (HOMO – LUMO) and energy gap (E_gap_) for the (*Z*)-1-[4-(Trifluoromethyl)benzylidene]thiosemicarbazide (In ground state) (isovalue = 0.02).

Experimental and theoretical computational data for both FTIR and ^13^C NMR spectroscopy are presented accordingly in Fig. 1, Fig. 2, Fig. 4, Fig. 5 to be used as complementary data for crystal of (*Z*)-1-[4-(trifluoromethyl)benzylidene]thiosemicarbazide [1] with CCDC:1507979 and were supported by other previous publications [2,3]. Both Fig. 3, Fig. 6 tabulates the correlation graph between experimental and theoretical computational data. In Fig. 7, Fig. 8 were presented the Molecular Electrostatic Potential (MEP) and Highest Occupied Molecular Orbital – Lowest Unoccupied Molecular Orbital (HOMO-LUMO), respectively. Whereas, Table 1 was calculated data derived from energy gap values using similar equation as previous reported [4].

**Table 1:** Calculated E_HOMO_, E_LUMO_, E_gap_, Electronegativity (χ), Chemical hardness (η) and softness (S) of (*Z*)-1-[4-(Trifluoromethyl)benzylidene]thiosemicarbazide.

E_HOMO_ (eV)	E_LUMO_ (eV)	E_gap_ (eV)	η (eV)	σ (eV)	χ (eV)
−8.6070	1.3778	7.2295	3.6146	0.2767	−3.6146

## 2 Experimental design, materials, and methods

### 2.1 Material

All chemicals and solvents were of analytical grade and were used as supplied.

### 2.2 Preparation of (Z)-1-[4-trifluoromethyl)benzylidene]thiosemicarbazide

A suspension of thiosemicarbazide (0.910 g, 0.01 mol) with 4-(trifluoromethyl)-benzaldehyde (1.740 g, 0.01 mol) was refluxed in ethanol (50 ml) for 5 hours. The white precipitate formed was filtered off and washed with cold ethanol. Finally, the precipitate recrystallized from hot ethanol, dried and kept in desiccator with silica gel.

### 2.3 Computational details

Optimized structure of (*Z*)-1-[4-trifluoromethyl)benzylidene]thiosemicarbazide was done with GaussView 5.0.9 and Gaussian 09 software package programme [5]. In theoretical studies, 6-311G (*d,p*) was selected as basic set due to standard theory level for C, H, N, S and F elements. Furthermore, the Density Functional Theory (DFT) method, named Becke, 3-parameter, Lee-Yang-Parr (B3LYP) was selected as method for studied their theoretical FTIR, ^13^C NMR, Molecular Electrostatic Potential (MEP), Highest Occupied Molecular Orbital (HOMO) and Lowest Unoccupied Molecular Orbital (LUMO) analysis in its optimized structure [6, 7]. Structure optimization was done at the minimum potential energy. Thus, all theoretical parameters were calculated at the minimum energy optimization.

MEP is useful to visualize variably charged regions of a (*Z*)-1-[4-trifluoromethyl)benzylidene] thiosemicarbazide molecule. Hence, the charge distributions can give the information about how the molecules interact with other molecules. In addition, determination the sites for electrophilic attack and nucleophilic reaction could be identified. Thus, the electrophilic reactivity is shown by the yellow regions and nucleophilic reactivity is shown by the blue region.

HOMO-LUMO determination and its several important key factors for conductivity activity which are the energy gap (ΔE_gap_), hardness (η), softness (σ) and the global electronegativity (χ) were calculated by using Eqs. (1), (2), (3), (4) as similar equation as reported, previously [4].

(1) $$\Delta {\text{E}}_{\text{gap}}={\text{E}}_{\text{LUMO}}-{\text{E}}_{\text{HOMO}}$$

(2) $$\eta =\frac{{\text{E}}_{\text{LUMO}}-{\text{E}}_{\text{HOMO}}}{2}$$

(3) σ=1/η

(4) $$\chi =-\frac{{\text{E}}_{\text{LUMO}}-{\text{E}}_{\text{HOMO}}}{2}$$
